# Lamp-Lit Bridges as Dual Light-Traps for the Night-Swarming Mayfly, *Ephoron virgo*: Interaction of Polarized and Unpolarized Light Pollution

**DOI:** 10.1371/journal.pone.0121194

**Published:** 2015-03-27

**Authors:** Denes Szaz, Gabor Horvath, Andras Barta, Bruce A. Robertson, Alexandra Farkas, Adam Egri, Nikolett Tarjanyi, Gergely Racz, Gyorgy Kriska

**Affiliations:** 1 Environmental Optics Laboratory, Department of Biological Physics, Physical Institute, Eötvös University, Budapest, Hungary; 2 Division of Science, Mathematics and Computing, Bard College, Annandale-on-Hudson, New York, United States of America; 3 Danube Research Institute, Centre for Ecological Research, Hungarian Academy of Sciences, Budapest, Hungary; 4 Group for Methodology in Biology Teaching, Biological Institute, Eötvös University, Budapest, Hungary; University of Western Australia, AUSTRALIA

## Abstract

Ecological photopollution created by artificial night lighting can alter animal behavior and lead to population declines and biodiversity loss. Polarized light pollution is a second type of photopollution that triggers water-seeking insects to ovisposit on smooth and dark man-made objects, because they simulate the polarization signatures of natural water bodies. We document a case study of the interaction of these two forms of photopollution by conducting observations and experiments near a lamp-lit bridge over the river Danube that attracts mass swarms of the mayfly *Ephoron virgo* away from the river to oviposit on the asphalt road of the bridge. Millions of mayflies swarmed near bridge-lights for two weeks. We found these swarms to be composed of 99% adult females performing their upstream compensatory flight and were attracted upward toward unpolarized bridge-lamp light, and away from the horizontally polarized light trail of the river. Imaging polarimetry confirmed that the asphalt surface of the bridge was strongly and horizontally polarized, providing a supernormal ovipositional cue to *Ephoron virgo*, while other parts of the bridge were poor polarizers of lamplight. Collectively, we confirm that *Ephoron virgo* is independently attracted to both unpolarized and polarized light sources, that both types of photopollution are being produced at the bridge, and that spatial patterns of swarming and oviposition are consistent with evolved behaviors being triggered maladaptively by these two types of light pollution. We suggest solutions to bridge and lighting design that should prevent or mitigate the impacts of such scenarios in the future. The detrimental impacts of such scenarios may extend beyond *Ephoron virgo*.

## Introduction

A taxonomically diverse array of nocturnal insects are attracted to artificial night lighting, where they can be captured by predators, die of exhaustion, or fail to locate suitable mates [1–4]. Altered species interactions triggered by light pollution can lead to declines in individual species [1,5] and overall biodiversity [6], as well trigger fundamental shifts in community composition that extend to daytime [7]. Less well-known is that strongly and horizontally polarized light reflected from artificial surfaces (e.g., asphalt roads, glass buildings, dark car-bodies) mimic the appearance of natural water bodies [8], attracting a broad range of aquatic insect taxa possessing and generally causing complete reproductive failure when eggs laid on these surfaces fail to hatch [9–11].

To date, the effect of this”polarized light pollution” [10] on aquatic insects has been studied exclusively during the daytime. Yet, artificial night lighting can also be polarized by reflection from asphalt and other man-made polarizers, creating strong nocturnal sources of polarized light that could attract night-active aquatic insects away from natural water bodies. Numerous case studies show that unpolarized light pollution and polarized light pollution can independently cause ecological traps [12] by creating misleading behavioral cues that attract insects away from their natural habitats [1,13–15]. Yet, the potential for these two different behavioral cues to interact remains unknown. And because ecological traps are capable of causing rapid population declines in affected species [16,17], the potential for two types of ecological traps to reinforce each other's effect is of high conservation concern.

**Fig 1:**
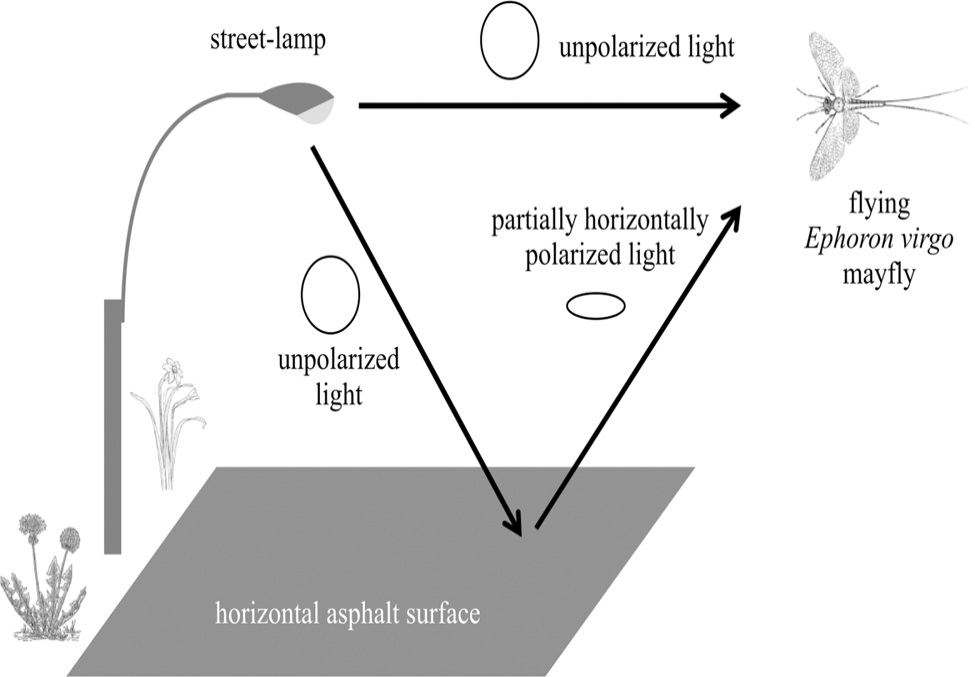
Nocturnal artificial sources of unpolarized and horizontally polarized light interact to attract polarotactic insects. Unpolarlized light sources (e.g., street-lamps) may attract perching or flying nocturnal polarotactic insects directly (*Ephoron virgo* mayfly illustrated). Alternatively, unpolarized light from the street-lamp can become horizontally polarized through reflection from smooth, dark surfaces like asphalt, simulating the appearance of a water body.

**Fig 2:**
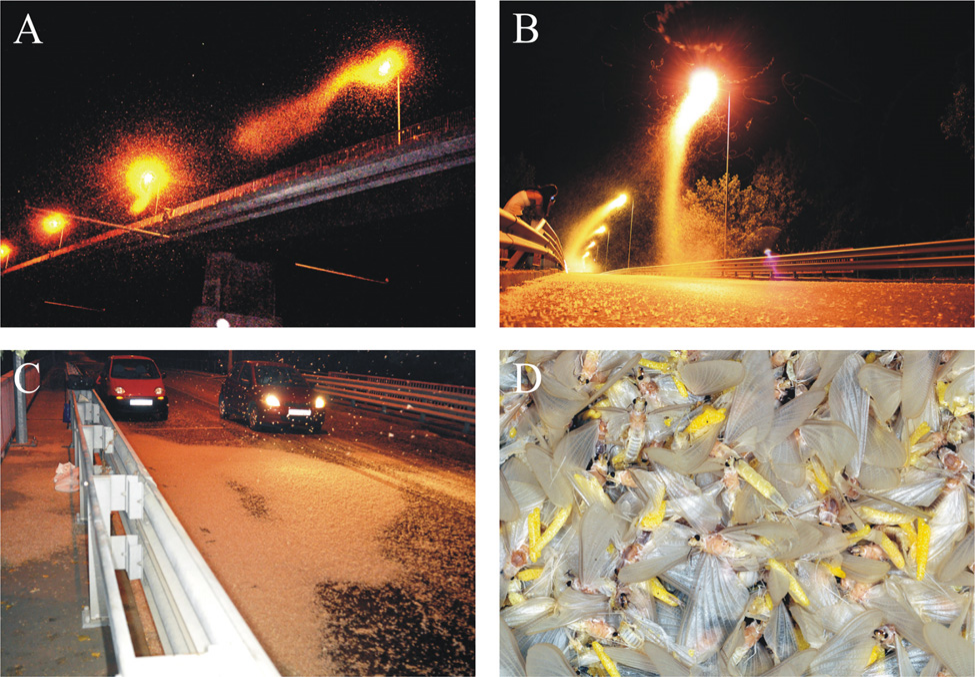
(A) Mass swarming of *Ephoron virgo* mayflies at night in Tahitótfalu (northern Hungary) at a bridge overarching the river Danube. (B) During the swarming we could observe that female mayflies performing their compensatory flight, flew up to the bridge-lamps. One part of females reaching the bridge landed on the asphalt road to oviposit, whereas the others joined to the swarm of several thousands individuals around the bridge-lamps. (C) With the progress of mass congregation the ovipositing females covered in greater and greater deal the asphalt road of the bridge. The mass of mayflies containing already perished and still ovipositing individuals formed large, extended, white stains. (D) The yellow egg batches are easy to recognise in the white crowd of mayflies, that consisted of several thousand eggs each.

High-intensity nocturnal lighting (e.g., street or security lights, stadium lighting) can produce wavelengths of unpolarized light that attract aquatic insects directly [4,13,18]. It is generally thought that this attraction is a maladaptive consequence of insects’ innate tendency to navigate using the light of the moon [1]. Most of these nocturnal light sources are commonly placed above asphalt-paved roadways and parking lots, the relatively smooth surface texture and dark color of which make them capable of polarizing reflected light with degrees of polarization *d* higher than about 60% at the Brewster angle [10,19]. Such high *d*-values are characteristic to natural dark waters [8,14]. Where such conditions occur in proximity to natural water bodies, insects originally attracted to unpolarized light pollution sources could, in theory, also find themselves confronted with a second false cue, horizontally polarized light, indicating the presence of a lake or river. A recent field experiment demonstrated that some aquatic insects were more attracted to lamp-lit artificial polarizers at night than unlit polarizing surfaces or unpolarized light sources without polarizers [20], suggesting that the interaction of polarized and unpolarized nocturnal light pollution should lead to the observation of this phenomenon in the wild. In this article we describe such a phenomenon observed and quantitatively documented in the field (Figs. 1 and 2).

*Ephoron virgo* is found throughout most of Europe, Turkey and North Africa. The burrowing larvae inhabit U-shaped tubes in the riverbed where they filter and eat suspended particles from the water current in the tubes by their abdominal gills [21,22]. Larval habitat mainly consists of sand [23] or gravel with sand and fine sediments [24]. The life cycle of *Ephoron virgo* is univoltine (one generation in a year) and characterised by a diapause egg stage from autumn to mid-April when the larvae hatch. The larval growing period lasts until August, when winged adult males and females emerge from the water and swarm for a few hours and die by the following morning [25]. The subimago is a sexually immature male metamorphic phenotype that emerges first, moves to riparian vegetation where it moults into the sexually mature adult male. Mature males return to the river forming aerial swarms (> ~300 individuals) above the water surface at about 19:30 h. As the swarming progresses, the number of copulating mayflies increases. Swarm densities increase between 19:40 h and 20:45 h. Females lay egg batches on the water surface *en masse* [21] dying soon after.

Anecdotal reports of mayfly mass congregations and maladaptive behavior near nocturnal lighting have motivated our research. Kureck [26] and Tobias [27] reported about large swarms of mayflies (*Ephoron virgo* and other species) around lamps along riversides and bridges, describing these incredibly dense aggregations as”summer snow drifts”: Mayflies were attracted in such masses (1.5 million individuals recorded in one night on an illuminated road surface) that the pavement below lamps was covered by a dense centimeter-thick layer of ovipositing adults. In the summers of 2012 and 2013, we observed the nocturnal mass swarming of *Ephoron virgo* at an asphalt-paved and lamp-lit bridge over the Danube river. This bridge forms an optical barrier, interrupting the upstream-directed compensatory flight of *Ephoron virgo* females by disrupting the reflection-polarization signature of the river below [28]. We also observed massive numbers of mayflies maladaptively laying eggs on the asphalt road of the bridge consistent with their typical behavior above naturally polarizing water bodies [19,29] into laying eggs onto the dry asphalt surface instead of the river.

Here we describe a series of observations and experiments that explain this phenomenon as a maladaptive behavioral response of mayflies to both unpolarized and polarized light pollution. We used imaging polarimetry to characterize the reflection-polarization patterns of various surfaces of the bridge and the river-section below. We conducted experiments to verify that *Ephoron virgo* are independently attracted to polarized and unpolarized light, and documented the spatial distribution and behavior of mayflies in relation to both types of light sources. Finally, we suggest a method how bridges and other lamp-lit littoral objects could be prevented from becoming optical traps.

## Results

### Mass congregation of mayflies

Between 15 August and 2 September in 2012 and 2013 we observed 13 and 10 mass swarmings of *Ephoron virgo*, in respective years, in warm and calm weather in Tahitótfalu (northern Hungary) at the bridge named after Zoltán Tildy (47° 75’ N, 19° 08’ E) overarching the river Danube (Fig. 2, S1 and S2 Tables). In this work a swarm is defined as the aggregation of at least 300 insects. Swarms did not occur on rainy, cool nights. According to our visual observations and videoclips, swarming mayflies often changed their flight direction, but remained constantly above the water surface elevating at most about 10 cm of height. The mayflies occupied the whole river surface except for a couple-of-meter-long lane next to the bank. The typical upstream flying compensational swarms appeared several meters above the water surface in the river mid-line and continued upstream along the Danube towards the bridge every warm night after 20:30 h (GMT + 2 h) (S1 Videoclip). Only females perform such compensational flight to compensate the downstream drift of eggs and larvae [30–33]. The compensatory-flying mayflies jammed in front of the bridge, but sooner or later they moved directly towards the bridge-lamps, or landed immediately on the asphalt road of the bridge (S2 Videoclip). Since the direction of compensatory flight always faces the flow direction of the river, flying females always approached the bridge from its southern side. This swarming above the river had finished every warm night at about 21:30 h.

Large mayfly swarms appeared only on sections of the bridge that were directly above the water. The bridge lamp directly over the water at the edge of the river attracted thousands of mayflies (based upon photographic evidence), while the adjacent bridge lamp, that was placed entirely over land, attracted fewer than 50 individuals and lamps located farther from the water attracted no mayflies (S1 Fig., S3 Videoclip). Masses of mayflies formed continuously swirling and bending “tails” starting at each bridge-lamp and elongated southward toward where a slight wind was blowing (Fig. 2A-B, S2 Fig., S4 Videoclip). Some of these tails touched the asphalt road, where several hundreds of *Ephoron virgo* (counted on photographs) landed on the asphalt surface and oviposited, dying soon after (Fig. 2C-D).

### Behavior of females

The majority of mayfly corpses covering the bridge surface was composed almost entirely of females (99.0 ± 0.03% = mean ± standard deviation, Fig. 2D). The majority of upstream-moving females executing their compensatory flight flew directly to the bridge-lamps, where they formed continuously growing swirling swarms (S2 and S4 Videoclips). However, a smaller proportion of compensatory-flying females landed immediately on the asphalt road, where they laid their egg batches and perished within about 15 minutes (S5 Videoclip).

**Fig 3:**
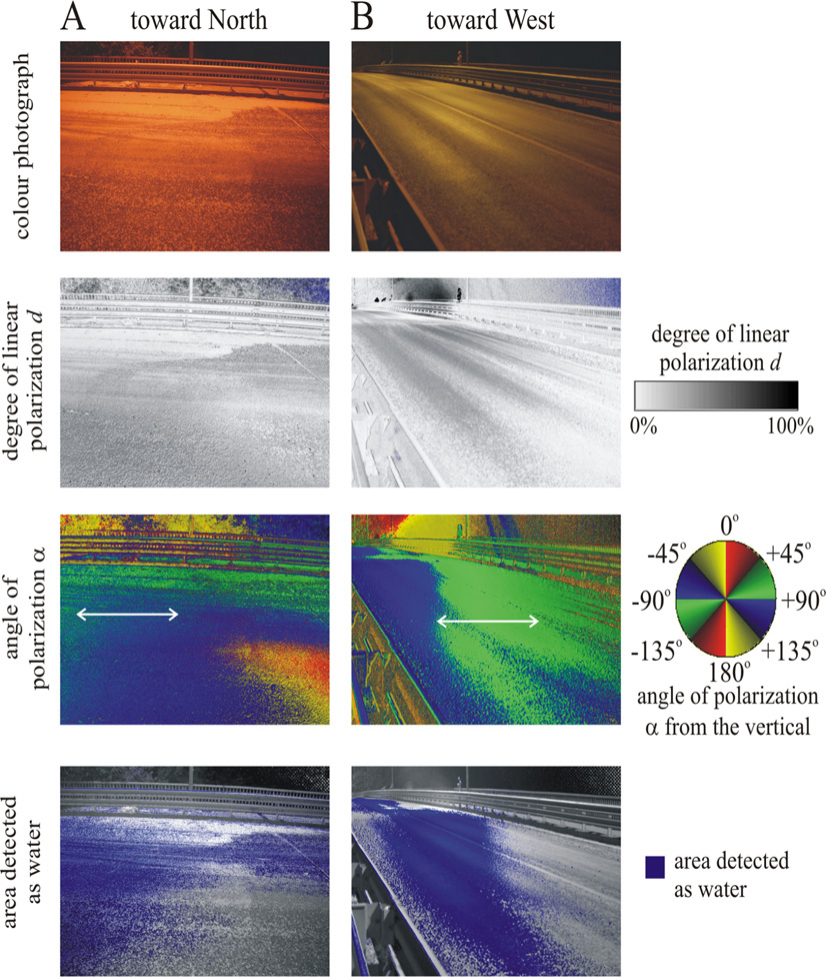
(A, B) Color photograph, patterns of the degree *d* and angle α (clockwise from the vertical) of linear polarization, and areas detected polarotactically as water (for which *d* > 15% and 80° < α < 100°) of the dry asphalt road on the bridge (above the river Danube at Tahitótfalu) illuminated by bridge-lamps at night during the mass congregation of *Ephoron virgo* mayflies. The patterns were measured by imaging polarimetry in the blue (450 nm) part of the spectrum from two different directions of view, when the optical axis of the polarimeter pointed toward North (A) and West (B). The angle of elevation of the optical axis of the polarimeter was 20° from the horizontal. In the α-pattern the local direction of polarization of asphalt-reflected light is shown by a double-headed arrow. The white spot composed of millions of *Ephoron virgo* carcasses on the asphalt road below the bridge-lamp is well visible on the photographs as well as in the patterns of the degree of polarization *d* and the area detected as water.

Mayflies moving upriver to encounter the bridge stopped arriving every night (when mass swarming occured) at about 21:30 h, thus swarms at bridge-lamps did not grow in size afterward. Swarming at bridge-lamps continued every night until 23:30 h. As swarming progressed, every night more and more females landed on the asphalt surface and laid their yellow egg batches, resulting in the coverage of the asphalt road by several-cm-thick layer of white mayflies and yellow egg batches in increasing areas (Fig. 2C-D, S6 Videoclip). Numbers of mayflies were so great that fluttering females, attempting to oviposit, landed on the top of layer of dead females which had already laid their eggs. This layer of white carcasses and yellow egg batches (Fig. 2C,D) depolarized the reflected light as seen in the *d*-patterns of Fig. 3, where the unpolarizing or only weakly polarizing asphalt regions are shown in white and light grey shades. This depolarization phenomenon practically eliminated the polarization signal of the asphalt surface. The depolarizing carcass layer was always the densest below the bridge-lamps, which suggests that beyond the polarization signal also the intensity of light was an important cue that governed the behavior of swarming females.

### Behavior of males

Male subimagos landed mainly on the light grey, matt, practically non-polarizing (*d* < 5%) concrete pavement running on the southern edge of the bridge and moulted to imagos (S3 Fig.). After moulting, the male imagos flew towards the bridge-lamps and joined the main mayfly swarms.

### Torch-light experiments

Flashlights attracted mayflies away from their flight paths both above the river and surrounding bridge-lamps. Upstream-moving individuals finished their compensatory flight, and gathered into the flashlight beam. Similarly, light beams directed toward swarms surrounding bridge-lamps attracted a significant number (several hundreds counted on photographs) distracted from the swarm into beams where they remained, moving to follow redirections of the beam. Once torch-lights were turned off, swarms associated with torch-light beams immediately broke up and the mayflies rejoined their main swarms around the bridge-lamps (S7 Videoclip).

### Distribution of mayflies at bridge-lamps

In the second half of the mass swarming (21:30–23:30 h), the elongated tail of the mayfly swarm circling around the bridge-lamps often reached the asphalt road. Individuals nearest to the asphalt surface were more likely to settle and oviposit on the asphalt than those near the lamp (S5 Videoclip). Live ovipositing individuals and dead females having already oviposited were concentrated directly below bridge-lights (Fig. 2C,D).

### Behavioral responses to polarized light sources

**Fig 4:**
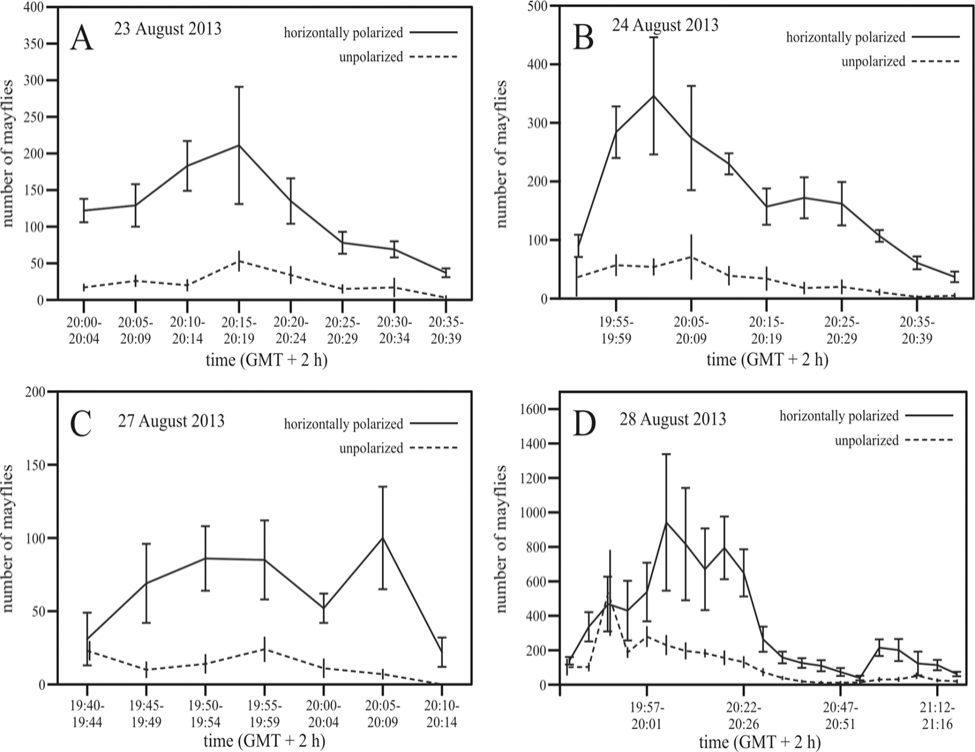
Estimated numbers of *Ephoron virgo* mayflies attracted to polarized and unpolarized light sources placed above the Danube river on four dates in 2012. Number (mean ± standard deviation) of mayflies attracted to horizontally polarized (continuous line) and unpolarized (dashed line) light as a function of time (= local summer time = GMT + 2 hours, where GMT = Greenwich Mean Time). Each estimate is based on 10 photographs (see subsection Experiments with linear polarizers of the Materials and Methods). Comparisons of total detections over the course of each test indicate that more individuals were attracted to polarized versus unpolarized light sources of the same intensity: (A) 23 August, N = 11582, U = 181, Z = 10.302, p < 0.00001; (B) 24 August, N = 22786, U = 674.5, Z = 11.388, p < 0.00001; (C) 27 August, N = 5425, U = 303.5, Z = 8.945, p < 0.00001; (D) 28 August, N = 93935, U = 8682, Z = 10.465, p < 0.00001, where N is the number of total mayfly detections, U is the parameter giving the sum of ranks used in the non-parametric method, Z is the standard deviation of data for a given p, and p is the level of significancy (p < 0.05 means significant).

We took 1500 photographs during four swarming days, identifying ~150000 individual mayflies using our image processing software. Mayfly abundance peaked between 19:50 and 20:20 h (GMT + 2 h). Depending on the swarming day, the horizontally polarized light attracted on average 5–10 times more mayflies than the unpolarized light (Fig. 4).

### Polarimetric measurements of the bridge and river surface

**Fig 5:**
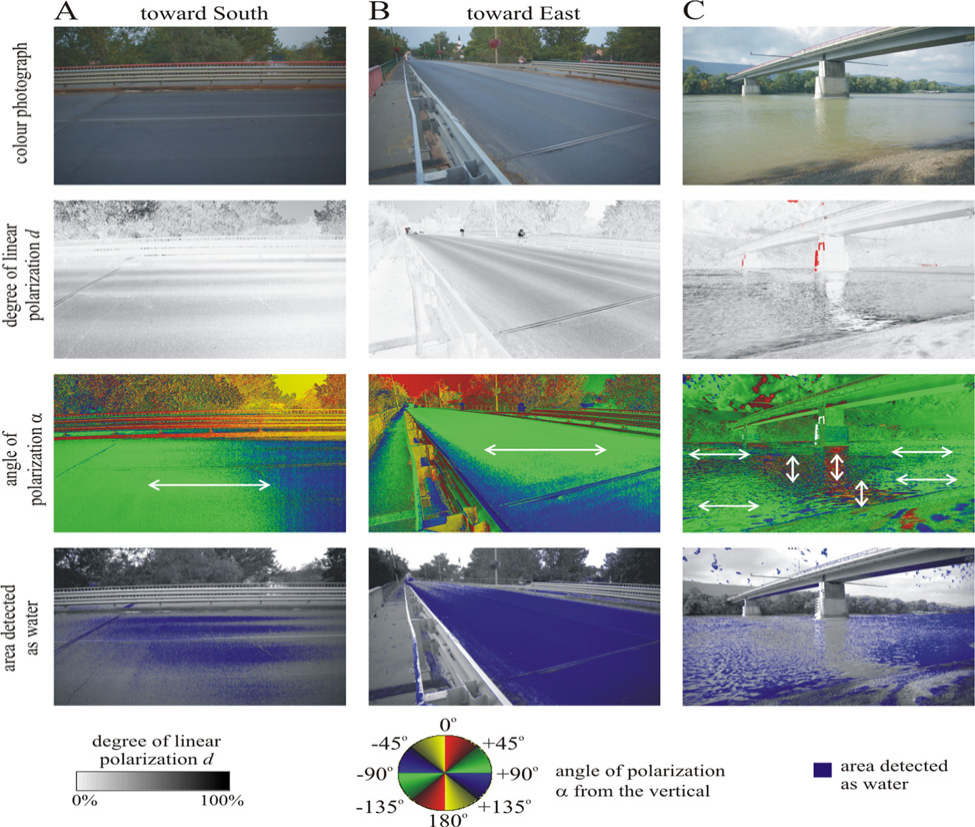
As Fig. 3 for the same asphalt road illuminated by light from the clear sky after sunset and measured from two different directions of view, when the optical axis of the polarimeter pointed toward South (A) and East (B). (C) As A and B for the down-stream side of the bridge and the Danube river.

The river surface reflected predominantly horizontally polarized light, with the exception of the parts with the shadow (in moonlight) and the mirror image (every night) of the bridge being only weakly (degrees of polarization *d* < 15%) and vertically polarized (Fig. 5). To mayflies approaching the bridge from downstream, this break in the horizontally polarized signature of the river will appear just downstream of the bridge itself, which reflects largely unpolarized (*d* < 5%) light (Fig. 5).

The asphalt road on the bridge reflects linearly (*d* > 20%) and nearly horizontally (angle of polarization clockwise from the vertical: 80° < α < 100°) polarized light from any direction before sunset (illuminated by skylight) and at night (illuminated by bridge-lamps) alike in all three (red, green, blue) spectral ranges (Figs. 3 and 5A,B) making it such detectable as water by polarotactic mayflies [9,19,29]. When mayflies landing on the asphalt road formed a thick, white, circle-shaped layer under the lamps (Fig. 2C), parts of the asphalt surface, which were covered by mayflies and their corpses, reflected entirely unpolarized (*d* = 0%) light (Fig. 3A, row 2: white and light grey marking; row 4: white marking).

## Discussion

We provide key evidence illustrating that (i) *Ephoron virgo* mayflies are independently attracted to both unpolarized and polarized light sources, (ii) both types of light pollution are being produced at a bridge, and (iii) the maladaptive swarming and oviposition of these mayflies can be explained by their positive phototaxis and polarotaxis. Previous experimental work [20], along with anecdotal observations of mass swarming near lighted bridges and other structures have suggested that polarized and unpolarized light pollution should interact in attracting insects toward unsuitable oviposition sites in the wild. Our results represent a case study providing evidence for the occurrence of such a scenario.

Previous field experiments have demonstrated that mayfly species detect water by the horizontal polarization of reflected light [9,19,29], and that this behavior is a ubiquitous evolutionary adaptation associated with nearly every aquatic and water-associated insect species [11,34–39]. During their ephemeral life as adults, horizontally polarized light compels both mayfly sexes to swarm and mate, and females to oviposit on the surface of the water, or the asphalt bridge surface in our study [14,19,28]. *Ephoron virgo* mayflies concentrated their oviposition activity where horizontally polarized light sources were generally strongest: most directly under the high-intensity bridge-lamps, the light of which was horizontally polarized after reflection from the asphalt road. Females avoided unpolarized portions of the bridge (e.g., concrete pylons and sides), less illuminated areas (portions of asphalt between bridge-lamps), and those portions that reflected vertically or obliquely polarized light (railings and walls). Furthermore, male subimagos emerging from the river were attracted to the bridge to molt, avoiding strongly polarizing asphalt in favor of light grey, matt, non-polarizing (*d* < 5%) concrete pavement (S3 Fig.) consistent with their preference for molting on riverbanks [24]. These observations suggest that positively polarotactic *Ephoron virgo* mayflies might have mistakenly perceived the asphalt road as a strongly and horizontally polarizing water surface.

Málnás *et al*. [28] found that the upstream-directed compensatory flight, that females of another local species (*Palingenia longicauda*) engage after metamorphosis and emergence from the water, is guided by females following the long, unbroken horizontally polarized light signature of the river. In that study, a bridge supported no artificial night lights, but blocked incoming skylight, creating a break in the continuous horizontally polarized light trail of the river and halting the compensatory flight of females. *Palingenia longicauda* females responded by forming a swarm above the water and downstream of the bridge and then ovipositing into the river. The bridge in our study created an identical break in the horizontally polarized light signature of the river, but *Ephoron virgo* females responded differently, moving vertically upward to first swarm near bridge-lights and above the bridge, then ovipositing on the asphalt road. Because *Ephoron virgo* very likely guides its compensatory flight using the same mechanism, attraction to the bridge surface in this species is an evidence that unpolarized street light pollution is causitive in attracting females away from the river surface.

This result also suggests that unpolarized light of high intensity can be a more attractive cue than water-polarized light in guiding the overwater compensatory flight. Yet, because the bridge-lights in our study were at a great height (about 20 m) over the water, there would be less light falling on the water surface and becoming horizontally polarized than if unpolarized light sources were in closer proximity to the river. Consequently, bridge height and night light intensity should alter the relative intensity of unpolarized versus polarized light cues to female mayflies approaching the bridge. In fact, our experimental work found, that near the water surface, more females were consistently attracted to horizontally polarized light sources, than to unpolarized lights, suggesting that females weight one cue more than another in guiding behavior. The relative importance of these two cues in triggering the evolutionary trap we document remain unclear, and may be contextualized to the compensatory flight and oviposition behaviors separately. Nevertheless, attraction to unpolarized bridge-lights in our study did not completely suppress the attraction of mayflies to asphalt-polarized light; females attracted to bridge-lights eventually oviposited on the road (S3 Videoclip). Collectively, and regardless of the relative importance of different light cues, evidence in our study is most consistent with a model of sequential attraction in which *Ephoron virgo* mayflies are initially attracted away from the water surface by high-intensity unpolarized light (Fig. 2A-B, S1 and S2 Figs.), and subsequently attracted to illuminated asphalt surfaces eliciting polarotaxis as polarized light traps (Figs. 2B,C and 3).

We measured the reflection-polarization characteristics of the river surface only in daylight due to the technical constraint that polarimetry performs poorly under the dim illumination conditions at night. Because both the degree and the angle of polarization of river-reflected light are independent of the intensity of incident light [8,35,39,40], our daytime polarimetric measurements were otherwise similar to those during the observed swarming of the nocturnal mayfly *Ephoron virgo*. Consequently, the daytime reflection-polarization patterns of the river surface (Fig. 5C) can be compared with the daytime (Fig. 5A,B) and night-time (Fig. 3) polarization patterns of the bridge studied. The intensity and spectrum of torch-light used in our experiment were different from those of the bridge-lights. Thus, the insects’ reactions to torch- and bridge-light are not directly comparable. Nevertheless, the torch-light experiment demonstrated that a light beam of relatively low intensity can attract mayflies even under the more intense bridge-lamps. This is an important practical result, which demonstrates that the mayflies attracted to the unpolarized bridge-lamps and polarized asphalt road can be protected by a light barrier composed of a series of smaller lamps with appropriate intensity and spectrum positioned at an appropriate height above the river surface: the flying mayflies are expected to gather in the beams of this protecting light barrier, rather than of the bridge-lamps.

Polarized light pollution can be produced in the absence of unpolarized nocturnal lighting where moonlight reflects from artificial polarizers (e.g., asphalt, solar panels, etc.), yet this kind of photopollution can only be as intense as the unpolarized light source, so, artificial polarizers illuminated by high-intensity street-lights will often be more attractive than those illuminated by the moon [20]. The emergence and activity of aquatic insects often decline when the moon is full [18,41,42] and so the attractiveness of the evolutionary trap we document may actually be mitigated during bright moonlit nights. In addition, high- and low-pressure sodium, metal-halide, and LED lights are all common forms of unpolarized nocturnal lighting that vary in the wavelengths of light they produce [4] and which affect their attractiveness to different aquatic insect taxa [43].

Light pollution is already known to have a broad range of negative impacts on animal populations and biodiversity, in general, with the ability to restructure and simplify freshwater food webs and alter the function of freshwater ecosystems [2,3,44] which, in our system, is compounded by the existence of an evolutionary trap. Evolutionary traps are cases in which, due to rapid environmental change, formerly reliable behavioral cues cause animals to preferentially behave in ways that reduce their fitness [45,46]. Because traps represent attractive population sinks, they can act to crash populations of affected species more rapidly than other causes of habitat degredation, mortality, or reproductive failure [17], making them a rapidly emerging conservation concern [12,45].

In general, eliminating evolutionary traps can accomplished by a) eliminating the attractive behavioral cues, b) increasing the fitness consequences of responding to cue(s), or c) both [12,47]. Because the suitability of asphalt roadways for mayfly reproduction cannot reasonably be accomplished, application of these principals to our bridge example generally precludes approach b (and, therefore, also c). Consequently, trap elimination must target the reduction and control of unpolarized and polarized light cues. Complete removal of lights from this bridge is a simple, obvious and comprehensive solution to eliminating this evolutionary trap. However, if bridge-lighting is necessary for purposes of driver safety, lights would likely attract fewer insects from the river surface if they were located closer to the road surface and shaded or otherwise directed downward toward the road and away from the river. An extra row of lights at the base of support pylons could preferentially attract insects away from the elevated road surface lighting and ensure they properly oviposit on water, but could attract predators which consume ovipositing females and create an evolutionary trap via a different mechanism [48,49]. Concrete roads and asphalt roads with a high gravel content are poor polarizers [40] and so resurfacing bridges with these materials would likely reduce or eliminate nocturnal polarized light pollution, but mayflies would still be drawn to unpolarized bridge-lights where they may experience enhanced predation and exhaustion and or captivity [13,44] and potentially distracting them from mating or eventually ovipositing [50].

Given the high number of eggs laid by ovipositing mayfly (Ephemeroptera) taxa in general, the bridge in our study (in isolation) seems unlikely to represent a threat to populations of *Ephoron virgo* in the Danube [22,51]. However, stretches of river with a higher proportion of similar riparian structures (especially in larger cities) may reduce reproductive success so dramatically via a wide-spread”vacuum cleaner effect” [3] that populations could decline. More importantly, the generality of aquatic insect attraction to horizontally polarized light [35,39] suggests that this dual-cue evolutionary trap will have similar impacts on other aquatic insect species, but could cause declines in taxa, the populations of which are smaller or whose life-history strategy makes them more susceptible to increased mortality or reproductive failure. And because attraction to linearly polarized light has evolved in many non-aquatic arthropod taxa as an orientational/navigational cue [35,39], nocturnal polarized light pollution may be a significant conservation problem even for entirely terrestrial arthropod taxa. Whether the negative demographic impacts of evolutionary traps such as these are sufficiently severe to reduce mayfly abundance in a river remains unclear. Demographic models that illustrate the potential for traps to reduce population growth in wildlife populations have been developed only for longer-lived vertebrates and empirical studies of any kind are entirely lacking.

## Materials and Methods

### Swarming behavior

We observed the mass swarming of *Ephoron virgo* in Tahitótfalu (northern Hungary) every evening between 15 August and 2 September 2012 and 2013 at the bridge named after Zoltán Tildy (47° 75’ N, 19° 08’ E) overarching the river Danube (Fig. 2). During the daily mass swarming on 1 September 2012 we collected individual mayfly specimens from the asphalt road of the bridge between 19:30 and 20:30 (= local summer time = GMT + 2 hours, where GMT is Greenwich Mean Time). The specimens were collected from the mass of dead individuals present on the asphalt road under the bridge-lamps. We collected 10 samples from under four neighboring bridge-lamps containing approximately 100 individuals each, the sex ratio of which was determined in the laboratory. We documented on which parts of the bridge the female mayflies landed and oviposited.

In 2012, during the periods of peak Mayfly abundance, we used hand-held flashlights (common torches) to experimentally test the effect of concentrated, collimated lightbeam on the movement and distribution of mayflies. We predicted that mayflies can be attracted to these portable light sources and that they would concentrate in the beam. This experiment was repeated on the river bank at a height of 1 meter above the water surface and 1 km distant from the bridge in an unlighted section. Standing on the bank, we pointed the lightbeam towards the mayflies performing their compensatory flight above the river mid-line and observed the behavior of the swarm.

### Estimating the swarming intensity of mayflies at bridge-lamps

We characterized qualitatively the swarming intensity of mayflies at bridge-lamps by a dual nomenclature: (i) Mass swarming means that at each bridge-lamp mayflies formed a 2–5 m long, continuously moving and bending tail starting the lamp and containing more than 50 individuals (e.g., the left bridge-lamp in S1 Fig.). (ii) Low swarming means that fewer than 50 mayflies were swarming at each bridge-lamp (e.g., the right bridge-lamp in S1 Fig.). We took photographs about the swarms at the bridge-lamps, and using our self-developed computer program (see Image Processing below), the number of mayflies (distinct bright spots) were counted.

### Imaging polarimetry

We measured the reflection-polarization characteristics of the river surface below the bridge overarching the observed river-section in the red (650 nm), green (550 nm) and blue (450 nm) spectral ranges on 23 September 2013 at a solar elevation that corresponded to that of the full moon previously in the swarming period. Thus, the circumstances of the illumination were similar and at the same time the high enough light intensity enabled to make polarimetric measurements that we could not have performed under the poor illumination at night. We also measured the reflection-polarization patterns of the asphalt road of the bridge before sunset (when incoming light from the clear sky illuminated the road) and at night (when only the bridge-lamps illuminated the road). The applied method of imaging polarimetry had been described in details previously [35,52].

Although in Figs. 3 and 5 we present only the reflection-polarization patterns measured in the blue part of the spectrum, we also measured these characteristics in the red and green spectral ranges. Due to the colorless (grey) feature of the asphalt road, the polarizing characteristic of the asphalt is practically independent of the wavelength of light. In Figs. 3 and 5 we chose the blue channel, because mayflies have ultraviolet- blue- and green-sensitive photoreceptors [53,54], and thus blue represents the middle of their wavelength sensitivity range. It is still unknown, in which spectral range *Ephoron virgo* and other mayflies sense the polarization of reflected light.

### Experiments with linear polarizers

Based on the preliminary torch-light experiment in 2012, we performed field experiments to examine the effect of light sources with different polarization characteristics to *Ephoron virgo* mayflies on 23, 24, 27 and 28 August 2013. We placed a LED (Light-Emitting Diode) torch of high intensity (UltraFire C8 Cree XM-L T6 LED) fixed to a tripod on the bank of the Danube river 110 m far from the bridge. Its beam of torch-light was pointed towards the swarm of mayflies performing their compensatory flight in the middle of the river upstream the flow direction. Although the actual bridge-lamps were normal incandescent lamps, nowadays more and more bridge-lamps are composed of modern LEDs. In this experiment we used a LED torch, because this kind of lamp produced a collimated beam of light with an intensity high enough to reach the compensatory-flying mayflies in the river mid-line.

In the first experiment, a horizontally polarizing filter was placed directly in front of the torch, thus it functioned as a horizontally polarized light source. The filter was composed of a depolarizing common white tracing paper and a linear polarizer (diameter = 15 cm, thickness = 1 mm, type: XP42-18 from ITOS, Mainz, Germany), the latter being outside, that is farther from the torch. In the second experiment we applied a filter in which the order of the tracing paper and the linear polarizer was reversed, thus the emitted light was practically unpolarized with the same intensity as that of the horizontally polarized light in the first experiment. We verified with imaging polarimetry that the tracing paper depolarized the torch-light effectively: the degree of linear polarization *d* of torch-light was smaller than 5% (S4 Fig.) which cannot be perceived by the polarization-sensitive mayfly species studied unil now [35,39]. Although the polarization sensitivity threshold d* of *Ephoron virgo* is unknown, it probably has a similarly high d*-value as other mayflies, such as *Baetis rhodani* (in the blue (450 nm) part of the spectrum: 32% ≤ d* ≤ 55%), *Ephemera danica*, *Epeorus silvicola*, *Rhithrogena semicolorata* (in the blue: 55% ≤ d* ≤ 92%), for example [29].

We conducted both experiments from the time a swarm performing compensatory flight above the river mid-line was formed until its cessation. The longest time interval lasted from 20:15 to 21:20 (GMT + 2 h) and shortened over the course of our study and began and ceased earlier (by about 5 minutes) each day.

During each experiment, we took 10 photographs of the area under the light beam with digital cameras (Nikon D90 and D3200) to assess quantitatively mayfly responses. After a photograph was taken, we switched off the torch for five seconds, then after switching it on again, we waited another five seconds before a new photograph was taken. Thus, the mayflies from the swarm in the river mid-line could reach the beam. We repeated this 10-second procedure before taking each photograph. After switching off the torch, the torch-light-attracted individuals rejoined the main mid-line swarm and flew forward to the bridge-lamps. Thus, we could photograph expectedly new individuals each time and therefore minimize pseudo-replication. We conducted both 100-second experiments four times at the same place of the riverbank alternating between the polarized and unpolarized stimulus from the formation of the compensational swarm to its cessation.

### Image processing

We took 1500 photographs (resolution: 3430x2278 pixels) and scaled the images to 80% of their original size for evaluation with a self-written computer software, called AlgoNet [55], which is able, among others, to design image processing algorithms and to execute them. For this work we wrote a program that automatically counts individual mayflies on photographs: Our algorithm identifies and seperates concentrations of white pixels (blobs) associated with *Ephoron virgo*: In these photographs the lamp-lit white mayflies were clearly visible in front of the dark (practically black) night background (S5 Fig.) that enabled to use semi-automated image processing for counting the number of mayflies in the light beam. Since field objects with similar colors may also be recognized falsely using a color-based algorithm, we used a mask that was not evaluated by the software (marked with light grey in S5 Fig.). The pattern of intensity *I* in the green (550 nm) channel of the photographs was converted to a binary black (*I* < I*) and white (*I* > I*) image by applying an appropriate threshold of I* = 50, where the maximum intensity of a pixel in the green channel was *I*_max_ = 255. In the black-and-white binary images contours of blobs were found by the method of Suzuki and Abe [56], which scans the binary image line-by-line, and then each line pixel-by-pixel until it finds a border. The algorithm determines whether the found border is of outer or inner manner (if there is a hole/void in a blob, for example). The software stores the information about this border (i.e. whether it is a black-white or a white-black transition in the binary image during the scan), then continues the scan until a new border is found. After scanning the whole image, the areas within the outermost borders are considered as blobs.

In our study, only those blobs were kept for further analysis which had an area between 4 and 10000 pixels. Finally, overlapping blobs and blobs close to each other (their distance being less than 20 pixels) were merged to form a single blob. The distance of two blobs was defined by the Euclidean distance of their centers, where the blob center was obtained using its image moments [57]. The final number of blobs in a given photograph gave the number of identified mayflies in the light beam. We tested the efficacy of this software on 20 photographs containing numerous (about 100–1000) mayflies: we counted visually the mayflies, then these photos were evaluated by the software. The difference between the visually and computationally determined numbers of recognized mayflies was lower than 5%.

### Statistical analysis

Using Mann-Whitney U tests, we compared the numbers of mayflies attracted to horizontally polarized light and unpolarized light. The non-parametric Mann-Whitney U test was chosen for statistical evaluation, because the distribution of the measured data (the numbers of attracted mayflies) differed so much from normal distribution, that conventional parametric tests could not have been applied without a data transformation, which could have falsified the result of the test.

### Field study permits/approvals

For the location and activities of our field study no specific permissions were required.

### Animal Ethics Statement

Our field work (involving visual observations, documentations via videoclips and photographs, optical experiments using polarized and unpolarized light sources only for attracting mayflies) did not perish any living individual of the *Ephoron virgo* mayfly. The mayfly specimens collected from the investigated bridge (to determine their sex ratio) were carcasses of dead insects.

## Supporting Information

S1 Fig Spatial variation in the distribution of mayfly swarms at bridge-lamps. Near the river’s edge, lamps closer to the Danube (left side in the picture) attract several thousands of mayflies, whereas at the neighboring lamp farther from the river (more inland, right side in the picture) only a few tens of *Ephoron virgo* were swarming.
(TIF)

S2 Fig Photographs of swarming *Ephoron virgo* mayflies at one of the lamps of a Danube bridge with increasing magnification from A to D. The mass of mayflies starting at the lamp and elongating in the blow direction of the dominant slight wind continuously changed its shape. The swarm consisted of several thousands of individuals estimated by our computer program.
(TIF)

S3 Fig Photographs of a male subimago of *Ephoron virgo* mayfly moulting to imago (A: start, D: end) on the light grey matt concrete pavement of a Danube bridge. The subimago is a metamorphic phenotype of the sexually immature male.
(TIF)

S4 Fig Polarizing (left) and depolarizing (right) filters used for field experiments with *Ephoron virgo* mayflies. The light transmitted through the filter on the left in each picture/pattern is totally linearly polarized *(d* = 100%), that of on the right is practically unpolarized (*d* < 5% which is not perceived by any known polarization-sensitive animal). In the α-pattern double-headed arrows show the direction of polarization of filter-transmitted light, which is horizontal and tilted at 45° from the horizontal for the left and right filter, respectively. The polarization patterns were measured by imaging polarimetry in the red (650 nm), green (550 nm) and blue (450 nm) parts of the spectrum.
(TIF)

S5 Fig Massive attraction of *Ephoron virgo* mayflies to a horizontally polarizing light source set on a tripod on the bank of Danube river. The light grey mask covers the area excluded from quantitative evaluation (counting of flying white mayflies) in order to avoid false mayfly recognition by the evaluating software.
(TIF)

S1 Table Swarming intensity of *Ephoron virgo* mayflies between 15 August and 2 September 2012 estimated on the basis of photographs taken at four bridge-lamps. Mass swarming: at each lamp mayflies formed a 2–5 m long, continuously moving and bending tail containing more than 50 individuals (e.g., the left bridge-lamp in S1 Fig.). Low swarming: fewer than 50 mayflies were swarming at each lamp (e.g., the right bridge-lamp in S1 Fig.). Mayflies were counted on the photographs with the use of our self-developed computer program.
(DOC)

S2 Table As S1 Table for 2013. (DOC)

S1 Videoclip The compensatory flight of *Ephoron virgo* female mayflies above the mid-line of river Danube. (WMV)

S2 Videoclip The compensatory-flying mayflies jammed in front of the bridge, and smaller swarms separated moving toward the bridge-lamps. (WMV)

S3 Videoclip The mass swarming of *Ephoron virgo* appeared only at the bridge sections that were above the river or above the riverside contacting the water. (WMV)

S4 Videoclip The majority of upstream-flying females executing their compensatory flight flew directly to the bridge-lamps, where they formed continuously growing and swirling swarms. (WMV)

S5 Videoclip Egg-laying *Ephoron virgo* females on the asphalt road of the bridge. (WMV)

S6 Videoclip As mayfly swarming progressed, more and more females landed on the asphalt surface and laid their yellow egg batches, resulting in the coverage of the asphalt road by several-cm-thick layer of white mayflies and yellow egg batches in gradually increasing areas. (WMV)

S7 Videoclip Flashlight of a torch attracted mayflies away from their flight paths surrounding a bridge-lamp. (WMV)
