# Supplementary figures and images for: Lamp-Lit Bridges as Dual Light-Traps for the Night-Swarming Mayfly, Ephoron virgo: Interaction of Polarized and Unpolarized Light Pollution
Source: PLoS One. 2015 Mar 27;10(3):e0121194. doi: 10.1371/journal.pone.0121194 (PMC4376897; doi:10.1371/journal.pone.0121194)

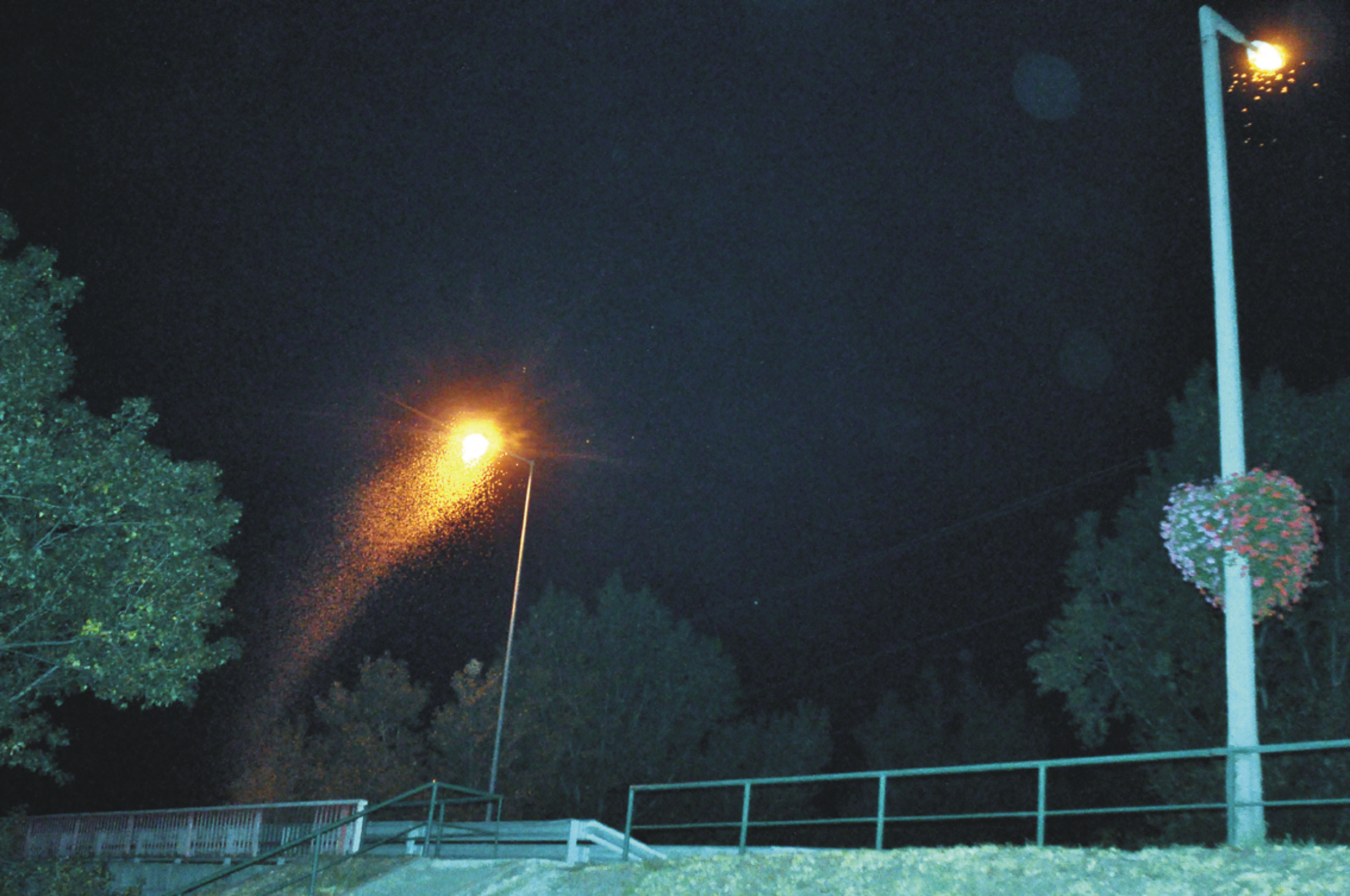

Supplement: S1 Fig — Near the river’s edge, lamps closer to the Danube (left side in the picture) attract several thousands of mayflies, whereas at the neighboring lamp farther from the river (more inland, right side in the picture) only a few tens of Ephoron virgo were swarming. (TIF) [file pone.0121194.s001.tif]

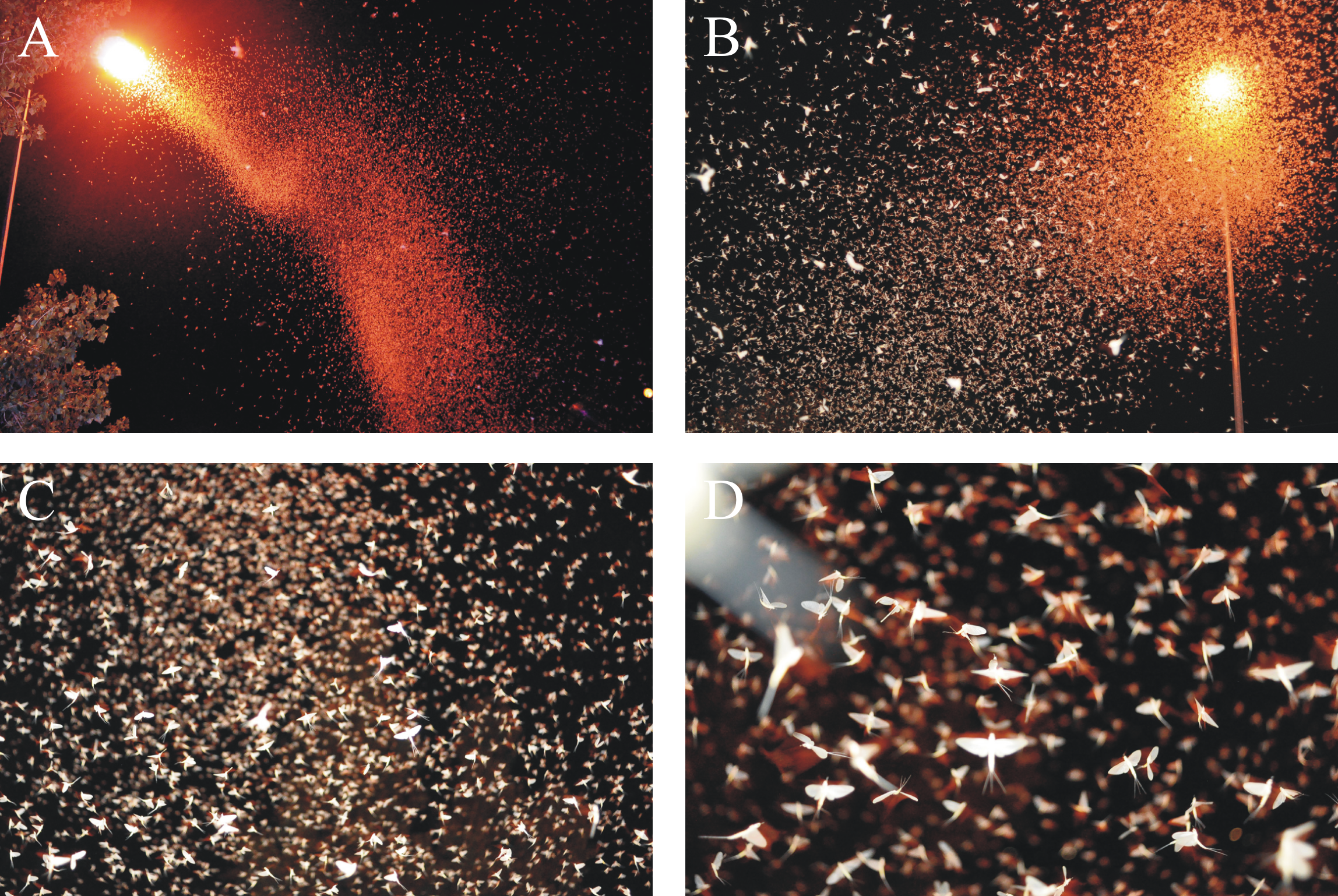

Supplement: S2 Fig — The mass of mayflies starting at the lamp and elongating in the blow direction of the dominant slight wind continuously changed its shape. The swarm consisted of several thousands of individuals estimated by our computer program. (TIF) [file pone.0121194.s002.tif]

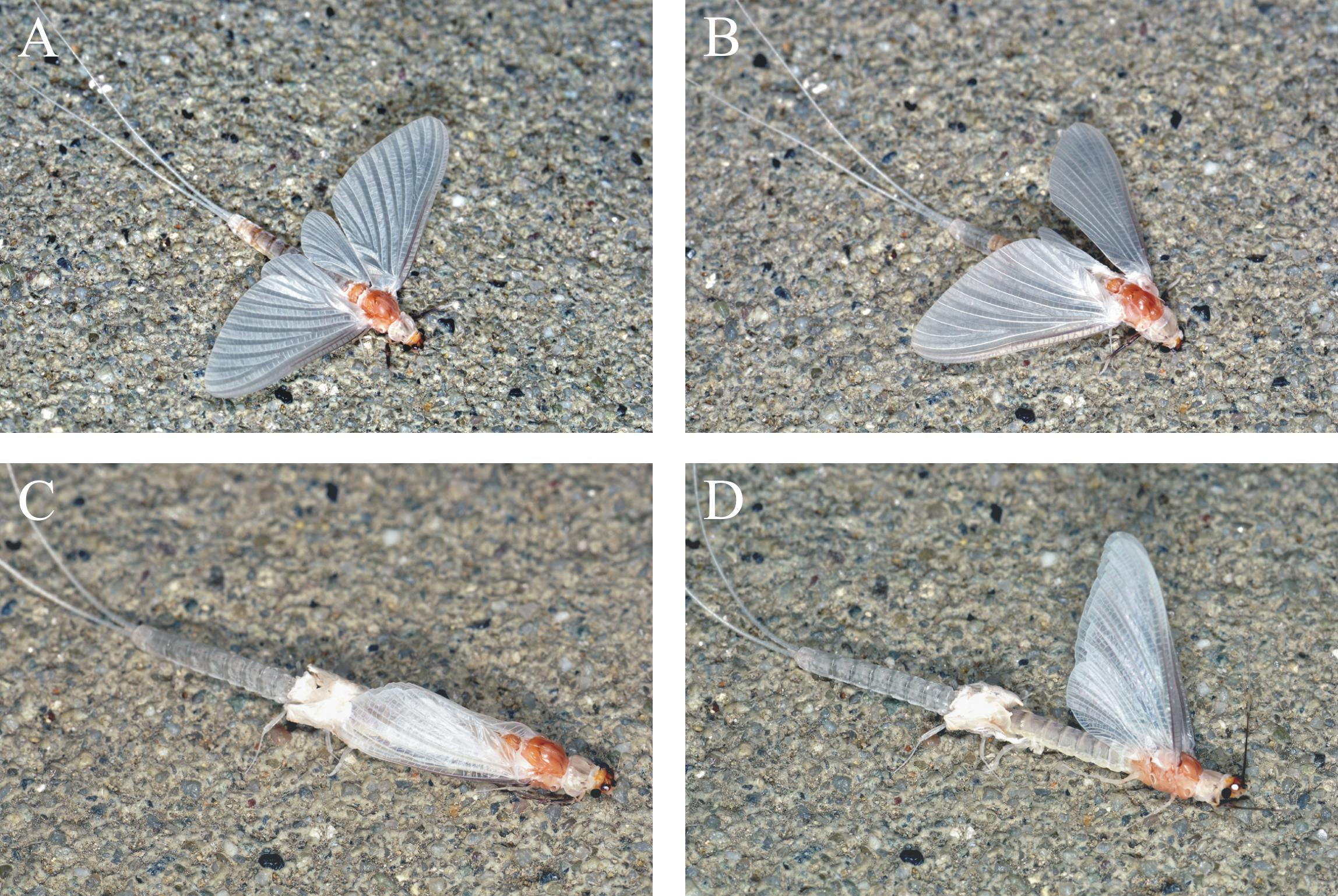

Supplement: S3 Fig — The subimago is a metamorphic phenotype of the sexually immature male. (TIF) [file pone.0121194.s003.tif]

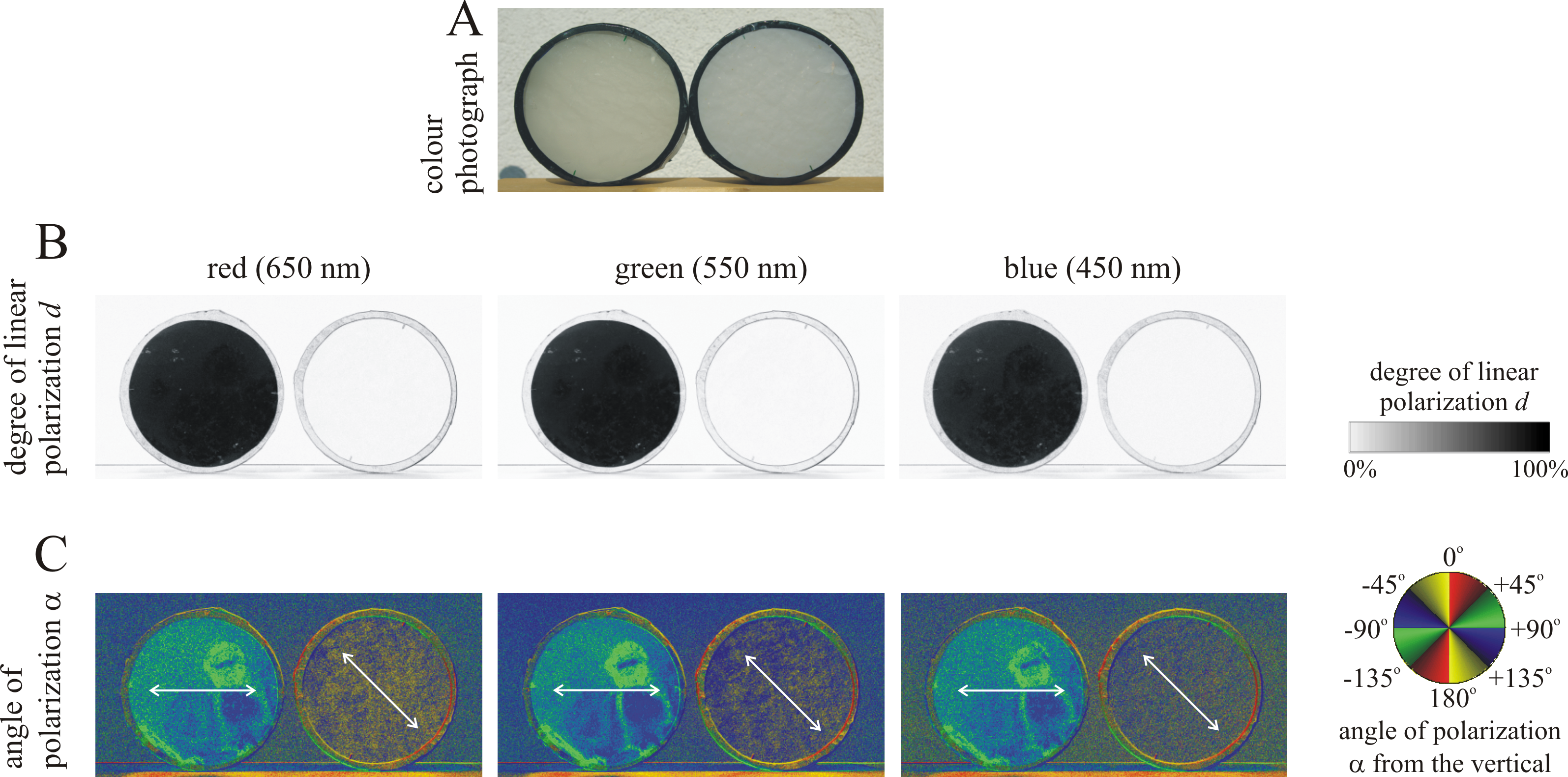

Supplement: S4 Fig — The light transmitted through the filter on the left in each picture/pattern is totally linearly polarized (d = 100%), that of on the right is practically unpolarized (d < 5% which is not perceived by any known polarization-sensitive animal). In the α-pattern double-headed arrows show the direction of polarization of filter-transmitted light, which is horizontal and tilted at 45° from the horizontal for the left and right filter, respectively. The polarization patterns were measured by imaging polarimetry in the red (650 nm), green (550 nm) and blue (450 nm) parts of the spectrum. (TIF) [file pone.0121194.s004.tif]

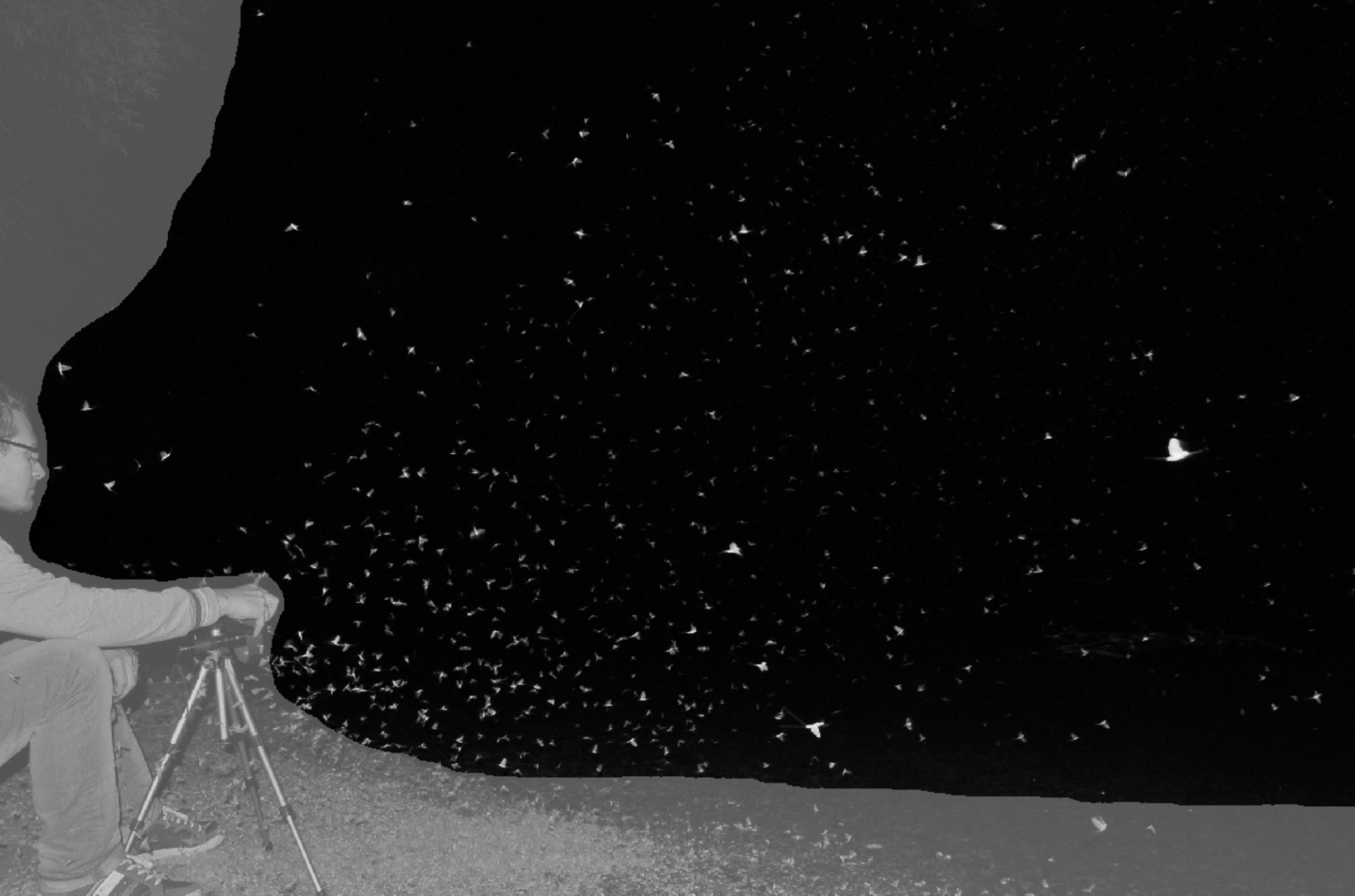

Supplement: S5 Fig — The light grey mask covers the area excluded from quantitative evaluation (counting of flying white mayflies) in order to avoid false mayfly recognition by the evaluating software. (TIF) [file pone.0121194.s005.tif]
