# Supplementary material for: Lamp-Lit Bridges as Dual Light-Traps for the Night-Swarming Mayfly, Ephoron virgo: Interaction of Polarized and Unpolarized Light Pollution
Source: PLoS One. 2015 Mar 27;10(3):e0121194. doi: 10.1371/journal.pone.0121194 (PMC4376897; doi:10.1371/journal.pone.0121194)
Supplement: S1 Table — Mass swarming: at each lamp mayflies formed a 2–5 m long, continuously moving and bending tail containing more than 50 individuals (e.g., the left bridge-lamp in S1 Fig.). Low swarming: fewer than 50 mayflies were swarming at each lamp (e.g., the right bridge-lamp in S1 Fig.). Mayflies were counted on the photographs with the use of our self-developed computer program. (DOC) [file pone.0121194.s006.doc]

**Table S1**

| **date**  **(2012)** | **swarming intensity of *Ephoron virgo*** | |
| --- | --- | --- |
|  | **mass swarming** | **low swarming** |
| 15 August |  | + |
| 16 August |  | + |
| 17 August |  | + |
| 18 August | + |  |
| 19 August | + |  |
| 20 August | + |  |
| 21 August | + |  |
| 22 August | + |  |
| 23 August | + |  |
| 24 August | + |  |
| 25 August | + |  |
| 26 August |  | + |
| 27 August |  | + |
| 28 August | + |  |
| 29 August | + |  |
| 30 August | + |  |
| 31 August |  | + |
| 1 September | + |  |
| 2 September | + |  |
| **sum** | **13 mass swarming** | **6 low swarming** |
