# Supplementary material for: Lamp-Lit Bridges as Dual Light-Traps for the Night-Swarming Mayfly, Ephoron virgo: Interaction of Polarized and Unpolarized Light Pollution
Source: PLoS One. 2015 Mar 27;10(3):e0121194. doi: 10.1371/journal.pone.0121194 (PMC4376897; doi:10.1371/journal.pone.0121194)
Supplement: S2 Table — (DOC) [file pone.0121194.s007.doc]

**Table S2**

| **date**  **(2013)** | **swarming intensity of *Ephoron virgo*** | |
| --- | --- | --- |
|  | **mass swarming** | **low swarming** |
| 15 August |  | + |
| 16 August |  | + |
| 17 August |  | + |
| 18 August |  | + |
| 19 August | + |  |
| 20 August |  | + |
| 21 August | + |  |
| 22 August | + |  |
| 23 August | + |  |
| 24 August | + |  |
| 25 August | + |  |
| 26 August | + |  |
| 27 August | + |  |
| 28 August | + |  |
| 29 August |  | + |
| 30 August |  | + |
| 31 August | + |  |
| 1 September |  | + |
| 2 September |  | + |
| **sum** | **10 mass swarming** | **9 low swarming** |
